# Development and pilot evaluation of a clinic-based mHealth app referral service to support adult cancer survivors increase their participation in physical activity using publicly available mobile apps

**DOI:** 10.1186/s12913-017-2818-7

**Published:** 2018-01-16

**Authors:** Camille E. Short, Amy Finlay, Ilea Sanders, Carol Maher

**Affiliations:** 1 School of Medicine, Freemasons Foundation Centre for Men’s Health, University of Adelaide, Adelaide, Australia; 2 Alliance for Research in Exercise, Nutrition and Activity (ARENA), Sansom Institute, School of Health Sciences, University of South Australia, Adelaide, Australia

**Keywords:** Physical activity, Cancer survivor, Behaviour change, mHealth, Intervention

## Abstract

**Background:**

Participation in regular physical activity holds key benefits for cancer survivors, yet few cancer survivors meet physical activity recommendations. This study aimed to develop and pilot test a mHealth app referral service aimed at assisting cancer survivors to increase their physical activity. In particular, the study sought to examine feasibility and acceptability of the service and determine preliminary efficacy for physical activity behaviour change.

**Methods:**

A systematic search identified potentially appropriate Apple (iOS) and Android mHealth apps. The apps were audited regarding the type of physical activity encouraged, evidence-based behavioural strategies and other characteristics, to help match apps to users’ preferences and characteristics. A structured service was devised to deliver the apps and counselling, comprising two face-to-face appointments with a mid-week phone or email check-up. The mHealth app referral service was piloted using a pre-post design among 12 cancer survivors. Participants’ feedback regarding the service’s feasibility and acceptability was sought via purpose-designed questionnaire, and analysed using inductive thematic analysis and descriptive statistics. Change in physical activity was assessed using a valid and reliable self-report tool and analysed using paired t-tests. In line with recommendations for pilot studies, confidence intervals and effect sizes were reported to aid interpretation of clinical significance, with an alpha of 0.2 used to denote statistical significance.

**Results:**

Of 374 mHealth apps identified during the systematic search, 54 progressed to the audit (iOS = 27, Android = 27). The apps consistently scored well for aesthetics, engagement and functionality, and inconsistently for gamification, social and behaviour change features. Ten participants completed the pilot evaluation and provided positive feedback regarding the service’s acceptability and feasibility. On average, participants increased their moderate-vigorous physical activity by 236 min per week (d = 0.73; 95% CI = −49 to 522; *p* = 0.09).

**Conclusion:**

This study offered initial evidence that a mHealth app referral service for cancer survivors is feasible and acceptable and may increase physical activity levels. The large increase in physical activity is promising, but should be interpreted with caution given the small sample size and lack of control group. Further research is warranted on a larger scale to investigate generalisability, long-term compliance and application in clinical settings.

**Electronic supplementary material:**

The online version of this article (10.1186/s12913-017-2818-7) contains supplementary material, which is available to authorized users.

## Background

The number of cancer survivors in the western world is steadily increasing [1]. This is owing in part to high incidence rates, and in part to advancements in detection and treatment which have led to improved survival outcomes. In Australia, survivors experience some of the highest survival rates in the world. The majority of those diagnosed (67%) now have a good chance of surviving for the next 5 years relative to their peers without a cancer history [2]. While increases in survival are duly welcomed, many survivors will suffer from adverse side effects well beyond the treatment phase, including fatigue, reduced physical functioning, premature aging, and mood disturbances [3, 4]. These issues have a significant and lasting impact on quality of life [5–7]. In addition, cancer survivors are at an increased risk of morbidity and premature death from cancer and non-cancer causes compared to age matched controls [8]. As such, cancer is increasingly recognised as a chronic disease, and cancer survivors as an at-risk group in need of rehabilitation and health promotion support [4].

One of the most effective rehabilitation strategies available is the participation in regular physical activity [9, 10]. There is strong evidence that physical activity can address the physical and mental sequelae associated with cancer and its treatment, and that it may also slow the progression of some cancers and reduce the likelihood of recurrence [11–14]. In recognition of these benefits, physical activity guidelines for cancer survivors have been published by professional bodies internationally [15, 16], and encouraging physical activity is recognised as an important component of on-going follow-up care [17]. However, translation of this evidence into physical activity services that are effective, accessible and sustainable has not yet been achieved. Unfortunately, most survivors do not have access to targeted support and most remain insufficiently active to achieve recovery and other health benefits [18].

Mobile health (mHealth) applications have been put forth as a promising intervention modality in this context [19–25]. They can offer convenient access to physical activity advice and support, an enjoyable user experience, and provide feedback on progress over-time [26]. Further, a recent review of 23 behaviour change mHealth interventions found that 17 of the evaluated mHealth apps produced a significant effect on health behaviours in the general population [23]. This is also reflected in cancer specific research, with preliminary studies suggesting that mHealth apps are feasible to deliver, are acceptable to survivors and may be efficacious [22, 24, 25]. However, in practice, finding good quality physical activity apps and maintaining engagement with them can be difficult. There are hundreds of physical activity related apps available in app stores, which vary considerably in their focus, approach to behaviour change and overall quality [27]. There is also research to suggest that different types of apps (e.g., a yoga app versus a walking app), or different app features (e.g., gamification, goal-setting, self-monitoring) may work better for some people more than others [28–31]. For example, individuals who enjoy competition and/or have a positive attitude towards games may engage more readily with a gamified app than those who would prefer a less social or more serious application [28, 29]. Overall, it is recommended that a person-based approach be adopted when providing apps to individuals, ensuring that the design and focus of the app accommodates the preferences and needs of the user [32]. Recent studies among cancer survivors suggest that this may necessitate the use of different apps for different people, especially when utilising existing mHealth apps [19–22].

We believe that a possible approach for addressing these factors may be an app selection and counselling service designed specifically for cancer survivors, which could be delivered by an appropriate health professional. This may reduce the confusion associated with selecting an app, increase the likelihood that the app is evidence-based and a good fit for the individual, and present an opportunity to provide additional support likely to increase engagement and efficacy. This is supported by reviews showing interaction with a counsellor increases adherence to digital interventions [33], and that digital interventions tailored to individuals are more effective than one-size fits all interventions [34]. The aims of the current study were three-fold. First, we aimed to develop a mHealth app referral service that is based on evidence and can be utilised in a typical health consultation session (e.g., with a physiotherapist or cancer nurse). To the best of our knowledge, no such services have previously been developed and evaluated. Second, we aimed to assess the feasibility and acceptability of the service among a mixed group of cancer survivors, and third examine the potential for efficacy for promoting physical activity.

## Method

This pilot study involved two key phases: Phase 1 focused on development of the mHealth app-based referral service, which included performing an audit of potential mHealth apps for inclusion in the referral service and devising the structure of this service. Phase 2 focused on piloting the mHealth app referral service with cancer survivors. The project was approved by the University of South Australia Human Research Ethics Committee, and all participants in the pilot study provided written informed consent.

### Phase 1. Development of the mHealth app referral service

#### Identification of publically available apps

**Fig. 1:**
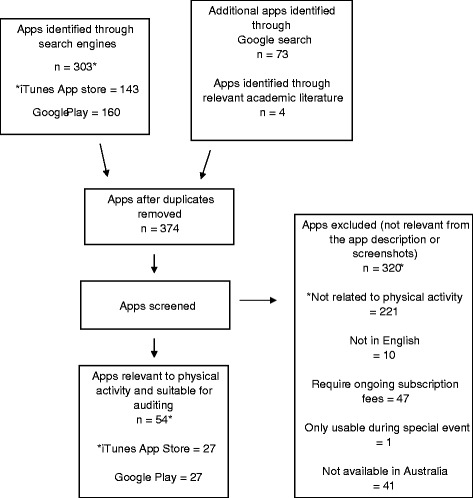
Flow chart of app screening process

To identify publically available mHealth apps, searches were conducted in the Australian Apple App Store, Google Play Store and via the Google search engine in August 2016. Apps on the first page in the “Health and fitness” category of both app stores were screened, as well as apps identified via searching using the terms “cancer”, “breast cancer”, “bowel cancer” and “prostate cancer” in the search window. Breast, bowel and prostate terms were searched specifically owing to the high prevalence of these cancers [1, 2]. For the Google search engine, the search term “cancer physical activity apps” was used. Google Scholar was also used to identify apps mentioned in previous literature, and these were then searched for in both app stores [35]. This was to ensure all previously evaluated apps that are publically available were identified (since these can be easily buried in the app stores). Apps were eligible for inclusion if they focused on physical activity in some capacity and were able to be downloaded onto an Android or Apple smartphone. Apps were excluded if they had on-going subscription fees, if they had not been updated since 2010, were not available in English, were directed at children, were designed for a special community event (e.g., fun run), or were unavailable in the Australian app stores. Apps did not need to be cancer specific to be included. Out of the 374 mHealth apps identified through searches, 54 were deemed eligible (27 iOS, 27 Android) and progressed to the audit stage (see Fig. 1).

#### Audit of mHealth apps

The apps were audited using an extended version of the ‘Mobile App Rating Scale’ [36]. The original scale, which has good internal consistency (alpha = 0.90) and interrater reliability (interclass correlation coefficient = 0.79), was extended to collect additional information considered important for recommending a mHealth app to cancer survivors. This included if the app required equipment (e.g., fitness tracker, weights), the type and dose of activity encouraged, the presence of evidenced-based behaviour change strategies [31, 37–41] and if the app contained features likely to be desirable based on user preferences and/or personality (e.g., social and gamification features) [28, 42]. The additional items were asked in accordance with previous app-audit studies [27, 35]. A pilot test of the Mobile App Rating Scale and the additional items was conducted independently by four members of the research team prior to commencing the audit on four randomly selected apps. A small number of discrepancies were identified (two differences in behaviour change techniques identified, and one difference in grade of functionality), and all were resolved by consensus. A complete copy of the audit tool is available via the study open science framework page (https://osf.io/m2zzh). Two research assistants (involved in the adaptation of the tool and initial pilot test) then reviewed all eligible apps using the tool (one focusing on Android apps and the other on iOS apps). An overall mHealth app quality score was calculated by first computing standardised sub-domain scores, and then summing them together. The six sub-domains included in the final score are listed in Additional file 1 with example items. To standardise the sub-domain scores, each sub-score total was divided by the total possible score and multiplied by 100. The scores were then reweighted so that each subscale contributed 16.66% of the total score. As such, the highest possible sub-domain score was 16.66 and the highest possible overall app quality score was 100. Higher scores signal higher app quality.

#### Development of the mHealth app referral matrix

To guide the recommendations of apps aligned with cancer survivors’ goals, preferences and needs, two app referral matrices were developed (for iPhone and Android users; see Additional files 2 and 3, respectively). This involved cross-referencing app quality, the features, foci and strategies used in the audited apps, with factors about the user likely to drive use, appreciation and effectiveness (e.g., willingness to pay for an app, preferred type of physical activity, workout goals, personality) [43, 44]. The aim was to a) establish a short-list of existing apps with reasonable quality that could cater to a heterogeneous sample of cancer survivors, and b) provide a quick reference guide for which people should receive which app, based on their individual characteristics.

#### Overview of the mHealth app referral service

A structured plan for the mHealth app referral service was devised by the research team. The service was intentionally designed to be minimalistic, so that it could be suitable for upscaling in future. The service was 1–2 weeks in duration, with an initial face-to-face consultation with a health counsellor, a mid-week telephone or email check-up, and a follow-up face-to-face session 1–2 weeks after the initial appointment with the same health counsellor. In this study, the health counselling was provided by two final year undergraduate physiotherapy students. In the initial session, a brief interview was conducted to assess the participant’s history and general health status (e.g., physical activity levels, aggravating factors, work status), physical activity interests (e.g., yoga, walking), app preferences (e.g., structured app with specific program vs. unstructured app that supports activity of your choice) and personality characteristics.

The mHealth app referral matrices were then used to select the most appropriate app for the participant. The remainder of the initial session involved supporting the participant to download the app and providing usage instructions. Participants also received education regarding the benefits of physical activity, the physical activity guidelines for cancer survivors [16], and a goal-setting activity, where participants were supported to specify their goal for the week (activity type, duration and frequency). Participants were provided with a single-page handout to record their goals and exercise sessions for the week [45]. The mid-week check-up was designed to identify and overcome any usability issues and to create a sense of accountability and support. If participants could not be reached by phone or if they preferred email, an email was sent. The follow-up session provided the participants with an opportunity to ask questions about the app and discuss any issues if present. During the session, current levels of physical activity were also reviewed and participants were assisted to create and write down longer-term goals (what they would like to achieve in 3 months’ time). Overall, each face-to-face session was 30–45 min in length. Telephone calls lasted no more than 5 min.

### Phase 2. Evaluation

#### Study design

The mHealth app referral service was evaluated using a single-arm pre-post-test design. Participants were assessed at baseline and immediately post-intervention using a questionnaire.

#### Setting and participants

The study was conducted at the Clinical Trials Facility of the Sansom Institute for Health Research at The University of South Australia between September and October 2016. Participants were recruited in a 1 month period using convenience sampling methods, including via social media and emails or telephone calls to research networks (e.g., South Australian Health and Medical Research Institute staff), consumer advocacy groups (e.g., Cancer Voices) and local cancer support groups. Potential participants were provided with information about the study and were instructed to contact the research team to express their interest in participating. Those who were over 18 years of age, who had previously been diagnosed with cancer, had completed primary curative treatment, and who had access to either an iPhone or Android smartphone were eligible. Recruitment materials noted that all participants would be reimbursed for their time at the end of the study with a $50 AUD visa debit card.

#### Measures

##### Participant characteristics

Participants’ demographic (age, gender) and health-related characteristics (cancer diagnosis, cancer treatments, self-rated health, co-morbidities) were collected at baseline.

##### Acceptability and feasibility

The extent to which the mHealth app referral service was acceptable and feasible to participants was assessed during the follow-up face-to-face session using a questionnaire comprising 24 purpose-built items based on previous research [46, 47] and theory relating to efficacy in online interventions [43]. Participants were asked to rate items on a 5-point Likert scale ranging from 1 – strongly disagree to 5 – strongly agree. Open-ended questions were also used to explore what participants liked and disliked about the service, and their recommendations for improvement.

##### Physical activity

Participation in aerobic physical activity was assessed pre- and post-intervention (i.e. in the first face-to-face session and again in the follow up face-to-face session, 1–2 weeks later) using the Active Australia Survey [48]. The survey assesses the duration and frequency of walking, moderate intensity and vigorous-intensity physical activity in the previous week. Total moderate-vigorous physical activity was calculated by adding the total time spent in each domain, with vigorous activity minutes weighted by two, in order to account for additional benefits [48]. The Active Australia Survey has acceptable test-retest reliability and validity in the Australian adult population (interclass correlation = 0.64; moderate correlation with objective step counts, Spearman correlation *r* = 0.42), and has been documented as a reliable and valid evaluative tool for detecting intervention related change in physical activity [49, 50]. Participation in resistance-based physical activity was assessed using a single additional item “do you currently do any resistance-based activities to improve your strength (e.g., light weights, resistance-bands)?” with a dichotomous response option (yes/no).

### Data analysis

Descriptive statistics were calculated for all study variables. Changes in participants’ aerobic physical activity levels from baseline to post-test were examined using paired sample t-tests. Changes in participants’ resistance-training behaviour were examined using Fisher’s exact test. These analyses were conducted in Stata 11 [51] on a complete case basis (*n* = 10). In line with recommendations for pilot studies, a type 1 error rate of alpha = 0.20 was used to denote significance [52]. Further, for aerobic activity confidence intervals and effect sizes were calculated to aid in the interpretation of results [52]. Effect sizes for aerobic activity changes were calculated using an online calculator [53]. Qualitative data were analysed using an inductive thematic analysis approach [54]. This approach is data-driven, and involves becoming familiar with the data, generating initial codes, searching for themes among codes and refining themes to better fit the data.

## Results

### Phase 1. Development of the mHealth app referral service

**Fig. 2:**
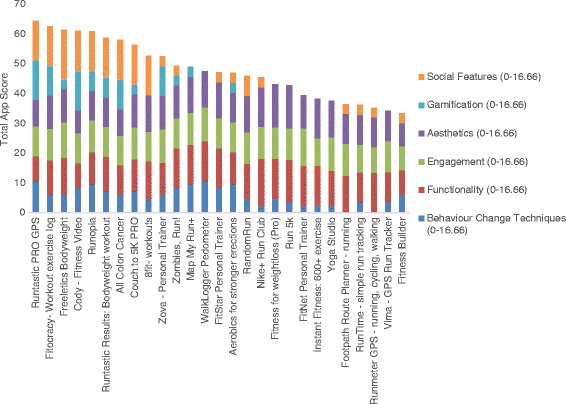
Sub-scale and total app scores for iPhone Apps

**Fig. 3:**
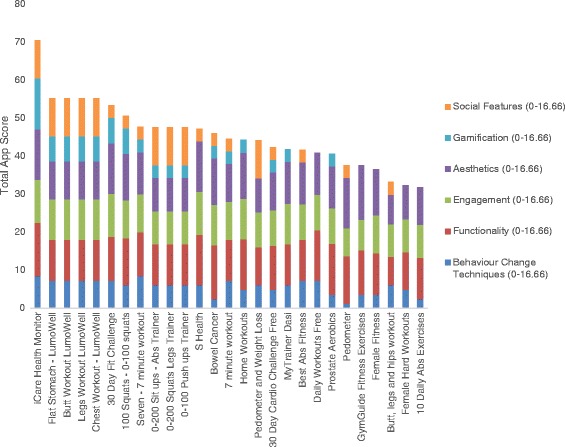
Sub-scale and total app scores for Android Apps

Out of the 374 mHealth apps identified through searches, 54 were deemed eligible (27 iOS, 27 Android) and progressed to the audit stage (see Fig. 1). Apps scores for iPhone and Android devices are reported in Figs. 2 and 3, respectively. The average app score was 46.81 (SD, 9.24) out of a possible 100. Scores ranged from 31.86 to 70.47. Overall, the apps consistently scored well in terms of their aesthetics, engagement features and functionality. However, there was wide variation in terms of the presence and extent of gamification and social features, and the use of accepted behaviour change techniques. Further, no apps designed specifically for physical activity promotion among cancer survivors were identified.

From the 54 mHealth apps audited, 30 unique apps were selected for use in the mHealth app referral service (15 iOS, 15 Android). The mHealth app referral matrices were used to select the minimum number of apps needed to address differing participant requirements. Higher scoring apps were selected where possible. However, as this involved cross-referencing app quality with desired features, focus and suitability for cancer survivors, not all high scoring apps were included and some moderate-low scoring apps were included. Known limitations of apps were considered when designing and delivering the mHealth app referral service to participants. The selected apps and the matrices used to deliver the service to iPhone and Android users are presented in Additional files 2 and 3, respectively.

### Phase 2. Evaluation

#### Participant flow

The flow of participants through the trial is presented in Additional file 4. Of the cancer survivors who expressed interest in participating (*N* = 17), five did not make an appointment for session one. The remaining participants (*n* = 12) were screened for eligibility and started the first session. Two participants withdrew from the study, one due to technical difficulties (the participant had an old phone model which was incompatible with all the recommended apps) and one due to an injury sustained while on holidays (unrelated to the study).

#### Participant characteristics

The average age of participants was 56 years (SD = 11.1), and ranged from 38 to 74. Six participants were female and four were male. Participants were survivors of breast cancer (*n* = 4), prostate cancer (*n* = 2), kidney cancer (*n* = 1), melanoma (*n* = 1), myeloma (*n* = 1), and non-Hodgkin lymphoma (*n* = 1). Average time since treatment was 6 years (SD = 4.6). Treatment with surgery (*n* = 8), radiotherapy (*n* = 8) and chemotherapy (*n* = 6) were commonly reported.

#### Acceptability and feasibility

##### Quantitative participant feedback

Mean scores for each item relating to acceptability and feasibility are provided in Table 1. Overall, participants agreed that the mHealth app referral service motivated them to do more physical activity (M = 4.20/5, SD = 1.03) and that it would be helpful to other cancer survivors (M = 4.40/5, SD = 0.69). The face-to-face sessions were found to be appropriate in terms of session duration (M = 4.30/5, SD = 0.48) and location (M = 4.20/5, SD = 1.03), and in terms of the explanation and guidance given about the app they were recommended (M = 4.40, SD 0.52). Recommended apps were generally considered easy to use (M = 4.10/5, SD = 1.10) and matching apps based on participant preferences was considered helpful (M = 4.20/5, SD = 0.79). Less positive appraisal was given for the duration of the service overall (participants would have preferred it to be longer, see Table 1), the usefulness of the mid-way phone or email contact (M = 2.80, SD = 1.48), and how enjoyable participants found the recommended app to use (M = 3.30; SD =1.25) (Table 1).

**Table 1:** Average response to acceptability and feasibility items (*n* = 10)

	Mean (SD)
Overall, the App Referral Service motivated me to do more physical activity	4.20 (1.03)^a^
Overall, the service has helped me feel more confident that I can engage in regular physical activity over the next 3 months	3.8 (1.03)
I found the duration of the service (1 week) suitable	3.1 (1.1)
Overall, the App Referral Service met my expectations	3.4 (1.17)
I would recommend the App Referral Service to other cancer survivors	4.1 (0.74)^a^
I think an App Referral Service will be helpful for cancer survivors upon completion of primary curative treatment to improve their physical activity	4.4 (0.69)^a^
Face-to-face sessions	
I found the physical activity information provided to me in the face to face sessions useful	3.8 (1.03)
I found the physical activity information provided to me in the face to face relevant to be personally	3.7 (1.16)
The questions used to assess my app needs and preferences in the first session were appropriate	4.5 (0.52)^a^
The interviews were of an appropriate duration (45 min – 1 h)	4.3 (0.48)^a^
The explanation and guidance given for the app was appropriate	4.40 (0.52)^a^
The location for the interviews was convenient for me	4.20 (1.03)^a^
The mid-way phone call/email	
The mid-way phone call/email helped to keep me accountable	2.80 (1.48)
The mid-way phone call/email helped to keep me motivated	2.80 (1.48)
The app recommended	
The app I was recommended is well suited to me preferences and needs	3.60 (1.08)
The app I was recommended is helping me to meet my personal physical activity goals	3.70 (0.95)
I found the app enjoyable to use	3.30 (1.25)
I plan to continue using the app to improve my physical activity	3.20 (1.62)
The app I was recommended met my expectations	3.70 (0.95)
I found the app I was recommended easy to use	4.10 (1.10)^a^
I have been actively using the app to try and improve my participation in physical activity	3.70 (1.33)
The type of exercises (e.g. walking, strength exercises, yoga, running, etc.) recommended to me through the app were well matched to my activity preferences	3.60 (1.08)
I found it helpful that the app was recommended according to my preferences	4.20 (0.79)^a^

^a^Scores of 4 or more indicate that on average participants agreed-strongly agreed with this item (possible range is from 1-strongly disagree to 5 – strongly agree)

##### Qualitative participant feedback

Six key themes were identified regarding what participants liked about the service. Namely, participants liked that the app recommendation was personalised, that the app provided was simple and easy to use, that the service was motivational, friendly and accessible, and that it was educational. While feedback was generally positive, most participants identified areas that could be improved and provided recommendations. Key areas for improvement included expanding the range of apps available so there are more activity options, and expanding the provision of information regarding app usage and the benefits of the service. Participants recommended that the service be provided for longer (e.g., include a 3 month follow-up), that custom apps specific to cancer survivors be on offer, along with an online version of the service. In addition, participants recommended that the service facilitate communication between health professionals and also the patients’ peers (e.g., via Facebook). Illustrative quotes relating to these findings are presented in Table 2.

**Table 2:** Illustrative quotes relating to participants’ experience of the app referral service (*n* = 10)

What did you like about the referral service?
Apps recommendations were tailored
*There’s so many apps out there, it’s really good for someone else to just sift through and target them*
*Consideration into choosing one for me*
*Personalised*
App simple and easy to use
*Easy*
*The app was simple and easy to use, especially for those who aren’t good at technology.*
*Able to check on steps easily. Easy to read without glasses. Not using my data – big plus*
*Found it easy to interpret exercise form app diagram*
*Simplicity*
Motivational
*Motivational*
*Overcome my barrier about starting daily skipping*
*Initial interviews provided start up motivation. Subsequent interview were good re-enforcement*
*Got me back on my bicycle*
*Motivated me to find app suitable to my exercise goals*
*Constructive*
Friendly and Accessible
*Friendly service and advice*
*Personable*
*Enthusiastic*
*Easy access and communication*
*I could connect to my T.V*
*Liked the app exercises*
Educational
*Good information*
*Well-presented and explained*
What could be improved?
Range and capabilities of apps
*Expand the range of apps*
*Frustrated with only small selection of activities provided. No weights provided on app*
*App developed specifically for cancer survivors*
More information
*Better explanation on use of apps, if person is not used to using apps.*
*Some more information about how this service can assist + support. Brochures of explanation*
What do you recommend?
Facilitate communication with professionals and peers
*Communicate with exercise physiologists/physio about individual risk factors patients may not understand*
*Examine non-technical, social basis approach which complement the app approach*
*Connect people that are participating in an online chat/Facebook group* etc.
*App referral service will be helpful for cancer survivors if in cooperation with guided personalised training*
Longer follow-up
*2–4 week use & follow up*
*Have the study be of longer duration*
*Two weeks would have been better to make it more habitual. Include a follow-up session after 3 months to see if people are maintaining their goals*
Targeted/tailored app specific to cancer survivors
*App developed specifically for cancer survivors*
*Closely monitored and tailored exercise programmes for cancer ‘rehab’ would be useful – taking into account levels of fatigue, lymphedema, peripheral neuropathy, etc*
Transfer service to online platform
Conduct interviews via skype or as an online form to fill out and submit

### Changes in physical activity

On average, participation in total weekly moderate-vigorous aerobic physical activity increased by 236 min from pre-test to post-test, which represents a moderate-large effect-size (Cohen’s *d* = 0.73). The finding was considered statically significant based on an alpha level of 0.20; 95% CI = − 49 to 522; *p* = 0.09). The greatest changes were seen for moderate-intensity physical activity (mean diff = 104 mins, 95% CI = −3, 211, *d =* 0.77, *p = 0.06*), followed by vigorous intensity physical activity (mean diff = 97 mins, 95% CI = −18, 213, *d = 0.41, p = 0.08*). Mean scores for pre- and post-test are presented in Table 3. At baseline, 6 participants were participating in some resistance-training. At follow-up, 7 participants reported participating in some resistance training. This increase was not statistically significant (*p* = 0.50).

**Table 3:** Physical activity scores pre and post intervention (*n* = 10)

	Baseline mean (SD)	Post-test mean (SD)	*P*-value	Cohen’s d
Total activity time (min)	358.50 (195.65)	594.70 (410.28)	0.09	0.73
Walking time (min)	187.00 (151.73)	239.50 (225.74)	0.49	0.27
Moderate activity time (min)	45.50 (64.83)	149.60 (180.61)	0.06	0.77
Vigorous activity time (min)	54.00 (95.71)	102.80 (137.37)	0.09	0.41

## Discussion

### Principle findings

Overall, the findings of this pilot study were encouraging. The mHealth app referral service was acceptable and was feasible to develop and deliver in a clinic setting, even by trainee health professionals. Further, the service had broad appeal to a diverse range of cancer survivors, and preliminary results suggest the service may help to support sizable physical activity behaviour change in the short-term. Some short comings of the service were identified, which should be addressed before wide-spread implementation is attempted, in order to maximise likelihood of success. The recommendations provided by participants, as well as findings from previous research, are useful in this regard.

### Suggestions for improving the service

In practice, it is likely that the service will need to be longer in duration to provide appropriate support. This was reflected in both the qualitative and quantitative process evaluation data in our study. This has also been reflected in previous research. A recent systematic review of interventions to improve diet and physical activity behaviours found that app-based interventions tended to be more effective when intervention periods were longer than 8 weeks [55]. Further, recent pilot studies among cancer populations suggest that interventions spanning 6-to-10 weeks are acceptable to users, at least in terms of engagement with the app and perceived usefulness of the intervention [19, 20]. However, fully-powered trials are needed to establish efficacy.

Given the diversity among cancer survivors, and their desire to receive programs tailored to their goals and needs [21], it may be prudent to explore the benefits and costs of an intervention with flexible delivery. It is well known that interventions with tailored content have greater efficacy than interventions with generic content [41]. It may be that interventions with tailored structures (e.g., flexible lengths and follow-up methods) are more effective than interventions with “one-size-fits all” structures. At least in theory, the best intervention length and structure will likely depend on the users’ goals as well as the type of app they are recommended.

A second suggestion for improving the service among our participants was increasing the range of mHealth apps available and/or having a cancer-specific app. This finding also resonates with previous research. A recent evaluation of survivors’ experience using a single freely-available app also found that a cancer-specific app was desired by some participants, and the use of a single app was unable to meet all participants’ needs [19]. Further, a qualitative study investigating survivors’ app preferences found that survivors desired an app that would give advice and feedback sensitive to their cancer diagnosis, personal health considerations, age, and location [21]. Our audit highlighted that, at present, cancer-specific apps are lacking. Until this gap is addressed, including further cancer-specific advice and support from the health counselling is warranted. Developing a cancer/specific app is also possible. However, whether or not a single app can be designed to provide relevant advice to this diverse user group, and do so in a way that is engaging remains unclear. An alternative approach may be to design a suite of mHealth apps that have a coherent behaviour change and engagement strategy for a particular type of user (e.g., someone motivated by competition), or for a particular kind of outcome (e.g., someone wanting to exercise to reduce cancer treatment symptoms). This may lead to more engagement in the long-term and thus better outcomes. This approach has been utilised in the mental health space with some success [56]. To do this well, however, and indeed to improve any kind of mHealth app referral service, more research into what apps are likely to work for who, as well as what app features work well together, is needed.

### Strengths and limitations

The current pilot study has several strengths. The intervention was rigorously developed and innovative in nature. Participation was not restricted to survivors of a specific cancer type, increasing the generalisability of results. Our findings provide new insights into how we may utilise existing mHealth apps to support cancer survivors’ physical activity. The preliminary evidence of efficacy for behaviour change was obtained using a widely-used physical activity measure with established reliability and validity. However, the magnitude of the change in physical activity was large, which may have been impacted by social desirability bias. In addition, whilst the mHealth app audit was conducted using an audit tool with established reliability and validity, we created a number of additional items to capture extra information deemed relevant to the current study, and we also created the acceptability and feasibility questionnaire. The psychometric properties of these questionnaire items are unclear. In addition, the study design did not include a control group, the sample size was small, and recruited using convenience methods. The intervention included a number of components (exercise counselling, variety of apps, follow-ups etc) so it is not possible to attribute affects to individual components. Further, outcomes were assessed using self-report data and long term follow-up was not conducted. Given the positive preliminary findings, further research evaluating this intervention approach on a large-scale, using a controlled trial designed, with longer-term follow-up and objective physical activity measurement is warranted. A mediation analysis of such a trial would also offer the opportunity to help understand the impact of individual intervention components.

## Conclusion

This novel study has provided preliminary evidence to suggest that an evidence-based mHealth app referral service is both acceptable and feasible for health professionals to provide tailored physical activity support for cancer survivors. This intervention was able to demonstrate its viability in a range of cancer survivors, and a moderate-to-large effect on physical activity behaviour was detected in the short-term. Based on these positive pilot findings, a larger RCT is warranted to determine long term efficacy of this tailored mHealth app referral service.

## Additional files

Additional file 1: Domains assessed and example items used in the adapted version of the Mobile App Rating Scale. (DOCX 15 kb)

Additional file 2: App Matrix for iPhone users. (DOCX 23 kb)

Additional file 3: App Matrix for Android users. (DOCX 22 kb)

Additional file 4: Figure showing flow of participants through the trial. (PDF 95 kb)

## Abbreviations

AUD: Australian dollars
Diff: Difference
iOS: Operating system for Apple mobile devices
mHealth: Mobile health
RCT: Randomised controlled trial
