# Supplementary material for: Development and pilot evaluation of a clinic-based mHealth app referral service to support adult cancer survivors increase their participation in physical activity using publicly available mobile apps
Source: BMC Health Serv Res. 2018 Jan 16;18:27. doi: 10.1186/s12913-017-2818-7 (PMC5771037; doi:10.1186/s12913-017-2818-7)
Supplement: Supplementary file 1 — Domains assessed and example items used in the adapted version of the Mobile App Rating Scale. (DOCX 15 kb) [file 12913_2017_2818_MOESM1_ESM.docx]

**Additional file 1: Table S1** Domains assessed and example items used in the adapted version of the Mobile App Rating Scale [28]

| **Domain** | **No. of Items** | **Raw Score range** | **Example item** |
| --- | --- | --- | --- |
| Use of evidence-based behaviour change techniques | 14 | 0-14 | Tailoring (i.e., a process of creating individual communications by gathering and assessing personal data and providing information or advice based on that data)  *1 if present, 0 if not present* |
| Functionality | 4 | 4-20 | Ease of use   1. *No/limited instructions; menu labels/icons are confusing; complicated* 2. *Useable after a lot of time/effort* 3. *Useable after some time/effort* 4. *Easy to learn how to use the app (or has clear instructions)* 5. *Able to use app immediately; intuitive; simple* |
| Engagement | 5 | 5-25 | Target Group (cancer survivors)   1. *Completely inappropriate/unclear/confusing* 2. *Mostly inappropriate/ unclear/confusing* 3. *Acceptable but not targeted. May be inappropriate/unclear/confusing* 4. *Well-targeted, with negligible issues* 5. *Perfectly targeted, no issues found* |
| Aesthetics | 3 | 3-15 | Visual appeal   1. *No visual appeal, unpleasant to look at, poorly designed. Clashing mismatched colours* 2. *Little visual appeal, poorly designed, bad use of colour, visually boring* 3. *Some visual appeal, average, neither pleasant or unpleasant* 4. *High levels of visual appeal, seamless graphics, consistent and professionally designed* 5. *As above plus very attractive, memorable, stand out, use of colours enhanced app features* |
| Gamification | 5 | 0-5 | Challenges (user opts in)  *1 if present, 0 if not present* |
| Social Features | 5 | 0-5 | There is an app community  *1 if present, 0 if not present* |
