# Supplementary material for: Development and pilot evaluation of a clinic-based mHealth app referral service to support adult cancer survivors increase their participation in physical activity using publicly available mobile apps
Source: BMC Health Serv Res. 2018 Jan 16;18:27. doi: 10.1186/s12913-017-2818-7 (PMC5771037; doi:10.1186/s12913-017-2818-7)
Supplement: Supplementary file 2 — App Matrix for iPhone users. (DOCX 23 kb) [file 12913_2017_2818_MOESM2_ESM.docx]

**Additional file 2 App Matrix for iPhone users**

| **App / Characteristics** | **Purpose of workout*** | | | | | **App function** | | **Willingness to pay** | | **Tech. self-efficacy** | | **Preferred exercise method*** | | | | | | **External device** | | | **Game** | | **Game characteristics** | | |
| --- | --- | --- | --- | --- | --- | --- | --- | --- | --- | --- | --- | --- | --- | --- | --- | --- | --- | --- | --- | --- | --- | --- | --- | --- | --- |
|  | **Increase running distance** | **Improve walking** | **General fitness** | **Race** | **Weight loss** | **Self-directed** | **Structured** | **Free** | **Paid** | **High** | **Low** | **Running** | **Cycling** | **Resistance** | **Yoga** | **Dragon boat** | **Walking** | **Required** | **No option** | **Optional** | **Game-based** | **Non game-based** | **Competitive** | **Cooperative** | **Social platform** |
| 8fit - workouts, meal planner & personal trainer |  |  | ✔ |  | ✔ |  | ✔ | ✔Paid upgrade option |  |  | ✔ |  |  | ✔ |  |  | ✔ |  |  | ✔Apple watch |  | ✔ | ✔ |  | ✔ |
| All Colon Cancer |  |  | ✔ |  | ✔ | ✔ |  |  | ✔$1.49 |  | ✔ |  |  | ✔ | ✔ |  |  |  | ✔ |  |  | ✔ |  |  |  |
| Instant Fitness: 600+ exercises |  |  | ✔ |  | ✔ | ✔ |  |  | ✔$4.49 |  | ✔ |  |  | ✔ |  |  |  |  | ✔ |  |  | ✔ |  |  | ✔ |
| Zova - personal trainer |  |  | ✔ |  | ✔ | ✔ |  | ✔Paid upgrade option |  |  | ✔ | ✔ |  | ✔ | ✔ |  |  |  |  | ✔Apple watch |  | ✔ |  | ✔Partner workout options |  |
| Fitocracy - workout exercise log |  |  | ✔ |  | ✔ |  | ✔ | ✔Paid upgrade option |  | ✔ |  |  |  | ✔ |  |  |  |  | ✔ |  | ✔ |  | ✔ | ✔ | ✔ |
| Nike+ Run Club | ✔ | ✔ | ✔ |  |  | ✔ |  | ✔ |  |  | ✔ | ✔ |  |  |  |  | ✔ |  |  | ✔Apple watch, fitbit, HR monitor |  | ✔ | ✔ |  | ✔ |
| Map My Run + GPS running | ✔ | ✔ | ✔ |  |  | ✔ |  |  | ✔$4.49 |  | ✔ | ✔ | ✔ |  |  |  | ✔ |  |  | ✔Apple watch, Fitbit, HR monitor |  | ✔ |  |  | ✔ |
| RandomRun | ✔ | ✔ |  |  |  | ✔ |  |  | ✔$2.29 |  | ✔ | ✔ |  |  |  |  | ✔ |  |  | ✔Apple watch |  | ✔ |  |  |  |
| Couch to 5k PRO | ✔ | ✔ | ✔ | ✔ |  |  | ✔ |  | ✔$4.49 |  | ✔ | ✔ |  |  |  |  | ✔ |  |  | ✔Apple watch |  | ✔ |  |  | ✔ |
| Fitnet - personal trainer |  |  | ✔ |  | ✔ | ✔ |  | ✔In-app purchases of extra workouts available |  |  | ✔ |  |  | ✔ | ✔ |  |  |  |  | ✔Apple watch |  | ✔ |  |  |  |
| Fitness for weight loss | ✔Running for weight loss app v. | ✔Walking for weight loss app v. | ✔ |  | ✔ |  | ✔ | ✔PRO v. for $4.49, no real advantage |  |  | ✔ |  |  | ✔ | ✔ |  |  |  |  | ✔  Fitbit |  | ✔ |  |  | ✔ |
| Yoga Studio |  |  | ✔ |  |  | ✔ |  |  | ✔$5.99 |  | ✔ |  |  |  | ✔ |  |  |  | ✔ |  |  | ✔ |  |  |  |
| FitStar - personal trainer |  |  | ✔ |  | ✔ | ✔ |  | ✔Paid upgrade option |  |  | ✔ |  |  | ✔ |  |  |  |  |  | ✔Apple watch, Fitbit |  | ✔ | ✔ | ✔ | ✔ |
| Runtastic results - bodyweight exercises |  |  | ✔ |  | ✔ | ✔ |  | ✔Paid upgrade option |  |  | ✔ |  |  | ✔ |  |  |  |  |  | ✔  Apple watch |  | ✔ | ✔ | ✔ | ✔ |
| Zombies, Run! | ✔ | ✔ | ✔ |  |  |  | ✔ | ✔Paid upgrade option |  |  | ✔ | ✔ |  |  |  |  | ✔ |  |  | ✔Apple watch | ✔ |  | ✔ |  | ✔ |
