# Supplementary material for: Development and pilot evaluation of a clinic-based mHealth app referral service to support adult cancer survivors increase their participation in physical activity using publicly available mobile apps
Source: BMC Health Serv Res. 2018 Jan 16;18:27. doi: 10.1186/s12913-017-2818-7 (PMC5771037; doi:10.1186/s12913-017-2818-7)
Supplement: Supplementary file 3 — App Matrix for Android users. (DOCX 22 kb) [file 12913_2017_2818_MOESM3_ESM.docx]

**Additional file 3** App Matrix for Android users

| **App / Characteristics** | **Purpose of workout*** | | | | | **App function** | | **Willingness to pay** | | **Tech. self-efficacy** | | **Preferred exercise method*** | | | | | | **External device** | | | **Game** | | **Game characteristics** | | |
| --- | --- | --- | --- | --- | --- | --- | --- | --- | --- | --- | --- | --- | --- | --- | --- | --- | --- | --- | --- | --- | --- | --- | --- | --- | --- |
|  | **Increase running distance** | **Improve walking** | **General fitness** | **Race** | **Weight loss** | **Self-directed** | **Structured** | **Free** | **Paid** | **High** | **Low** | **Running** | **Cycling** | **Resistance** | **Yoga** | **Dragon boat** | **Walking** | **Required** | **No option** | **Optional** | **Game-based** | **Non game-based** | **Competitive** | **Cooperative** | **Social platform** |
| **iCare Health Monitor** |  |  | ✔ |  | ✔ | ✔ | ✔ | ✔ |  | ✔ |  |  |  | ✔ |  |  |  |  | ✔ |  | ✔ |  |  |  | ✔ |
| **30 Day Fit Challenge Workout** |  |  | ✔ |  | ✔ |  | ✔ | ✔ |  |  | ✔ |  |  | ✔ |  |  |  |  | ✔ |  | ✔ |  |  |  | ✔ |
| **S Health** | ✔ | ✔ | ✔ |  | ✔ | ✔ |  | ✔ |  | ✔ |  | ✔ | ✔ |  |  |  | ✔ |  | ✔ |  |  | ✔ |  |  |  |
| **Pedometer & Weight Loss Coach** |  |  |  |  | ✔ | ✔ |  | ✔ |  | ✔ |  |  |  |  |  |  |  |  | ✔ |  |  | ✔ | ✔ | ✔ | ✔ |
| **Legs Workout LumoWell** |  |  | ✔ |  |  |  | ✔ | ✔ |  | ✔ |  |  |  | ✔ |  |  |  |  | ✔ |  | ✔ |  |  |  | ✔ |
| **Seven - 7 minute workout** |  |  | ✔ |  |  |  | ✔ | ✔ |  |  | ✔ |  |  | ✔ |  |  |  |  | ✔ |  | ✔ |  |  |  | ✔ |
| **30 Day Cardio Challenge Free** |  |  | ✔ |  |  |  | ✔ | ✔ |  |  | ✔ |  |  | ✔ |  |  |  |  |  | ✔ | ✔ |  |  |  | ✔ |
| **Bowel Cancer** | ✔ | ✔ | ✔ |  |  | ✔ |  | ✔ |  | ✔ |  | ✔ | ✔ | ✔ |  |  | ✔ |  | ✔ |  | ✔ |  |  |  | ✔ |
| **Butt, legs & hips workout** |  |  | ✔ |  | ✔ |  |  | ✔ |  |  |  |  |  | ✔ |  |  |  |  |  |  |  | ✔ |  |  | ✔ |
| **Pedometer** |  | ✔ | ✔ |  |  | ✔ |  | ✔ |  |  | ✔ |  |  |  |  |  | ✔ |  | ✔ |  |  | ✔ |  |  | ✔ |
| **MyTrainer Dasi** |  |  | ✔ |  | ✔ |  | ✔ | ✔ |  | ✔ |  |  |  | ✔ |  |  | ✔ |  | ✔ |  | ✔ | ✔ |  |  |  |
| **Home Workouts** |  |  | ✔ |  | ✔ |  | ✔ | ✔ |  |  | ✔ |  |  | ✔ |  |  |  |  |  | ✔ | ✔ |  |  |  |  |
| **Daily Workouts Free** | ✔ | ✔ | ✔ |  | ✔ |  | ✔ | ✔ |  |  | ✔ | ✔ |  | ✔ |  |  | ✔ |  |  | ✔ |  | ✔ |  |  |  |
| **Female Hard Workouts** |  |  | ✔ |  |  |  | ✔ | ✔ |  |  | ✔ |  |  | ✔ |  |  |  |  | ✔ |  |  | ✔ |  |  |  |
| **7 minute workout** |  |  | ✔ |  | ✔ |  | ✔ | ✔ |  | ✔ |  |  |  | ✔ |  |  |  |  | ✔ |  | ✔ |  |  |  | ✔ |
