# Supplementary figures and images for: Development and pilot evaluation of a clinic-based mHealth app referral service to support adult cancer survivors increase their participation in physical activity using publicly available mobile apps
Source: BMC Health Serv Res. 2018 Jan 16;18:27. doi: 10.1186/s12913-017-2818-7 (PMC5771037; doi:10.1186/s12913-017-2818-7)

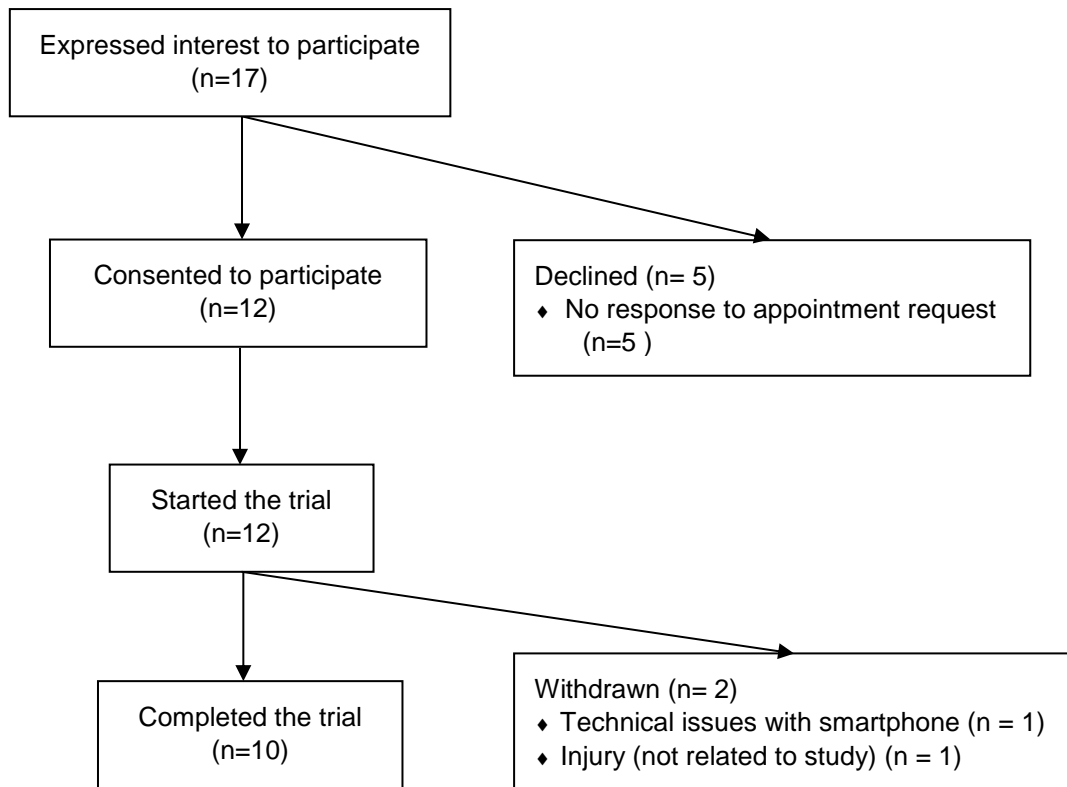

Supplement: Supplementary file 4 — Figure showing flow of participants through the trial. (PDF 95 kb) [file 12913_2017_2818_MOESM4_ESM.pdf]
